# Effectiveness of the Gamma Probe in Childhood Parathyroidectomy: Retrospective Study

**DOI:** 10.7759/cureus.6629

**Published:** 2020-01-11

**Authors:** Ozgur Caglar, Ibrahim Otgun, Hatice Yalcin Comert, Arzu Gencoglu, Esra Baskin

**Affiliations:** 1 Pediatric Surgery, Ataturk University, Erzurum, TUR; 2 Pediatric Surgery, Memorial Hospital, Ankara, TUR; 3 Pediatric Surgery, Karadeniz Technical University, Trabzon, TUR; 4 Nuclear Medicine, Baskent University, Ankara, TUR; 5 Pediatric Nephrology, Baskent University, Ankara, TUR

**Keywords:** secondary hyperparathyroidism, gamma probe detection, tc-99m-mibi, end-stage renal disease

## Abstract

Background

There are few reports about parathyroidectomy due to secondary hyperparathyroidism in patients with end-stage renal failure in the literature. We aimed to evaluate the surgical treatment methods and the results of patients who were operated for secondary hyperparathyroidism with end-stage renal disease in our center.

Method

Sixteen patients with the diagnosis of secondary hyperparathyroidism were treated surgically in our center. Demographical data, laboratory findings, and imagining methods were all examined. The effect of the Technetium 99m methoxyisobutylisonitrile (Tc-99m-MIBI) probe sensitive to gamma rays detection was also evaluated to locate and identify all the parathyroid glands during the operation.

Results

Eleven of the patients underwent intravenous (IV) Tc-99m MIBI preoperatively and a gamma probe was detected during surgery. The gamma probe was not used in five patients. Four parathyroid glands were removed in eight (72.7%) out of 11 patients with gamma probes and three parathyroid glands were found in three patients. Total parathyroidectomy and parathyroid autoimplantation were made to eight patients who had removed four glands, subtotal parathyroidectomy was done for the other patients. On a comparison of laboratory findings before and after the surgery, there was a significant relationship between the decrease of serum parathyroid hormone and calcium levels (p<0.05).

Conclusion

Total parathyroidectomy and parathyroid autoimplantation is the most efficient and safe mode of management for secondary parathyroidism patients. During the surgery, using a probe sensitive to gamma rays detection may also help the surgeon. Thus, unnecessary dissections to prevent the presence of atypical parathyroid glands are prevented.

## Introduction

Hyperparathyroidism occurs when parathyroid hormone (PTH) levels are increased as a result of the overwork of the parathyroid glands [1-2]. It is rarely seen in childhood. Clinical manifestations of hyperparathyroidism are nonspecific findings such as loss of appetite, weakness, restlessness, and abdominal pain due to hypercalcemia.

There are three different clinical types: primary, secondary and tertiary. Secondary hyperparathyroidism often occurs in cases of end-stage renal disease and, more rarely, rickets, osteomalacia, and intestinal malabsorption [2]. In secondary hyperparathyroidism, 1, 25-dihydroxy vitamin D3 production is impaired due to end-stage renal failure. In order to compensate for chronic hypocalcemia, the PTH level increases. PTH, which increases as a result of hyperplasia in parathyroid glands, mobilizes calcium from the bones, and increases serum calcium level.

The diagnosis of parathyroid hyperplasia is made by clinical, laboratory, and imaging methods. Ultrasonography (US) and parathyroid scintigraphy are often used for imaging methods [3-5]. In patients with hyperparathyroidism secondary to end-stage renal disease, calcium levels decrease as a result of a rapid decrease in PTH after parathyroidectomy. Parathyroid autotransplantation is performed to prevent this, and serum calcium levels are regulated [1,5-8].

It may be challenging to determine the localization of the parathyroid glands during surgery. The use of gamma probes during surgery has been becoming increasingly common in determining the location of parathyroid glands [9-11]. For this purpose, preoperative technetium-99m-methoxyisobutylisonitrile (Tc-99m-MIBI) is administered intravenously (IV) to detect the gamma probe during surgery. With this method, it is easier and faster to find the parathyroid glands, which are more difficult, especially in children. Thus, unnecessary dissections to prevent the presence of atypical parathyroid glands are prevented.

It has been seen in the literature that the number of cases with parathyroidectomy due to secondary hyperparathyroidism in children and adolescents is low, and it is mostly performed by adult surgeons [12-13]. The experience of adult surgeons does not fully reflect the treatment and follow-up methods related to children.

This study aimed to evaluate the diagnosis and treatment modalities of patients who were diagnosed with secondary hyperparathyroidism and end-stage renal disease and underwent parathyroidectomy.

## Materials and methods

A total of 16 patients who were diagnosed with secondary hyperparathyroidism by clinical and laboratory findings and imaging methods were included in the study. The study was approved by the medical ethics committee of our hospital (Project No: KA 10/80).

All patients were followed up by the pediatric nephrology department, diagnosed with secondary hyperparathyroidism, and planned as a treatment option with the pediatric surgery department. Surgical indications were parathyroid hormone (PTH) values above 700 pg/ml and those with clinical symptoms that did not respond to medical treatment with phosphate binders and active vitamin D analogs. The information of the patients was screened. The demographic data of the patients, the duration of follow-up period with the diagnosis of end-stage renal failure, diagnostic methods, the use of gamma probes during the operation, the state of parathyroid glands, and postoperative follow-up periods were recorded.

Serum calcium, phosphorus, parathyroid hormone, and hemoglobin levels were recorded preoperatively. In the postoperative period, calcium, phosphorus, and PTH values were also examined in the early postoperative (1 week) and late postoperative period (6 months). Calcium values before calcium replacement were considered postoperatively.

Preoperative parathyroid ultrasound (US) and parathyroid scintigraphy findings were recorded in all patients. Eleven patients who had a gamma probe as an adjunct to surgery were preoperatively given Tc99-m-MIBI (0.3 mCi/kg) and were screened by a gamma probe from all four quadrants during anesthesia induction. Gamma probe detection was performed from the sites considered to be the parathyroid gland during surgery, the counts were compared with the preoperative counts, and localizations of the parathyroid glands were determined. The counts of the removed lesion were taken before excision, and after the excision, the counts and the counts of the removed ground were recorded. When the parathyroid gland and the ground count were intermittent, it was thought that the value obtained was 1.5 mm in the areas with thyroid tissue on the ground and 2.5 mm in the non-thyroid gland.

Four parathyroid glands were removed and one-third of a parathyroid gland was implanted into the sternocleidomastoid muscle or subtotal parathyroidectomy was done. According to the surgeon's preference at the time of surgery, a Penrose or a hemovac drain was used or no drain was used.

Histopathological assay

Tissue samples from all the patients except the two patients underwent frozen examination. Thus, parathyroid tissue was confirmed histopathologically. All tissue specimens were evaluated histopathologically.

Statistical analysis

Statistical analysis of the data was done using the SPSS 15.0 package program (SPSS Inc., Chicago, Illinois). In the evaluation of data, the smallest, largest, and average values were used. Compliance with normal distribution was performed with the Kolmogorov Smirnov test. In the comparison of continuous data, the t-test was used in the case of normal distribution and the Mann-Whitney U test was used in the case of a not normal distribution. The Mc Nemar test was used in the dependent groups while the Kikare test was used in the independent groups and the Fisher Exact test was used in the insufficient number of groups. The significance level was accepted as 0.05 for all statistical tests used.

## Results

Of the patients, 11 (69%) were male and five (31%) were female and the mean age was 11.4±5.4 (1-18). The mean weight was 25.9±1.3 (7.5-37) kg. Preoperative hemoglobin values were found to be mean 8.7±1.7 (6.2-12.3) gr/dl, calcium values average 9.0±1.4 (10.6-5.2) mg/dl, phosphorus values average 7.3±2.2 (3.2-13.5) mg/dl, and PTH values were found to be 2145.9±910.9 (734-4540) pg/ml on average.

Radiology and nuclear medicine evaluation findings

Parathyroid US and parathyroid scintigraphy were performed preoperatively. It was found that 75% of patients had parathyroid pathology with a US, but 62.5% of them had been diagnosed with adenoma, 12.5% had only pathology in the parathyroid glands, and no distinction could be made between hyperplasia and adenoma.

With both examinations, 25% of the patients were reported to have no parathyroid pathology. Parathyroid pathology was detected by scintigraphy in two of four patients who were reported to have no parathyroid pathology with the US, but no differential diagnosis was made and the other two reported no pathology. When parathyroid US and scintigraphy were evaluated, parathyroid pathology was present in 14 of 16 patients and it was not detected in two patients.

Surgical findings

All patients underwent bilateral neck exploration with a Kocher incision. Eleven of the patients underwent IV Tc-99m MIBI preoperatively and a gamma probe was detected during surgery. A gamma probe was not used in five patients. Four parathyroid glands were removed in eight (72.7%) out of 11 patients with gamma probes and three parathyroid glands were found in three patients (Table 1). Four parathyroid glands were removed in one of five patients who had no gamma probes, and three parathyroid glands were found in four of them. A gamma probe was used in three (43%) of seven patients in whom all parathyroid glands could not be removed, and four (57%) were not used (Table 1). The contribution of gamma probes to the removal of all parathyroid glands was not statistically significant (p> 0.05). The use of a gamma probe and the removal of the parathyroid glands are shown in Table 1.

**Table 1:** Use of gamma probes and removal of parathyroid glands

Number of removed glands	Gamma probe usage status		Total
	No	Yes	
4 glands	1	8	9
3 glands	4	3	7
Total	5	11	16

Parathyroidectomy was performed in eight of the patients, and one-third of the parathyroid glands were implanted with the sternocleidomastoid muscle and parathyroid autotransplantation was performed. One patient underwent subtotal parathyroidectomy. In seven patients, three parathyroid glands were removed and no autotransplantation was performed. In the postoperative period, no complications, such as wound infection, hematoma, or recurrent laryngeal nerve injury, occurred.

The patients were followed up closely for signs of hypocalcemia in the postoperative period. IV Ca + 2 - gluconate (1 cc / kg / dose) was given to patients who developed hypocalcemia. The dose range was adjusted according to the patient's clinic and controlled serum calcium level. The patients needed IV Ca + 2 - gluconate for a mean of 8.6 ± 8.5 (1-30) days. Oral calcium lactate was added to the treatment after the patients were started on enteral feeding.

Patients who had a postoperative follow-up period of mean two to 10 (mean 4.5) days, starting from the day of surgery. After discharge, patients were followed-up for six to 74 (mean 18) months, with PTH levels, calcium, phosphorus, and other routine biochemical tests. In the late follow-up period, routine biochemical tests and PTH levels were also evaluated. In six of seven patients whose parathyroid glands could not be removed, PTH values were not sufficient and one patient decreased to 26 pg/ml.

Patients whose PTH levels were above the normal level were monitored because of decreased serum calcium levels and clinical signs of hyperparathyroidism. Because only two of these patients had hyperparathyroidism findings, it was decided to perform a second operation Only two of these patients were operated for the second time due to hyperparathyroidism findings that did not respond to medical treatment. One year after the first operation, with the help of a gamma probe, the fourth parathyroid gland was found and a mini-incision was made through the lesion. The other patient underwent bilateral exploration after seven years of the first operation, but no fourth parathyroid gland was found. In the last follow-up of both patients, no signs of hyperparathyroidism were detected. The other five patients with three parathyroid glands were observed without any need for a second operation because of the decline of their clinical findings.

Comparison of laboratory values

**Figure 1:**
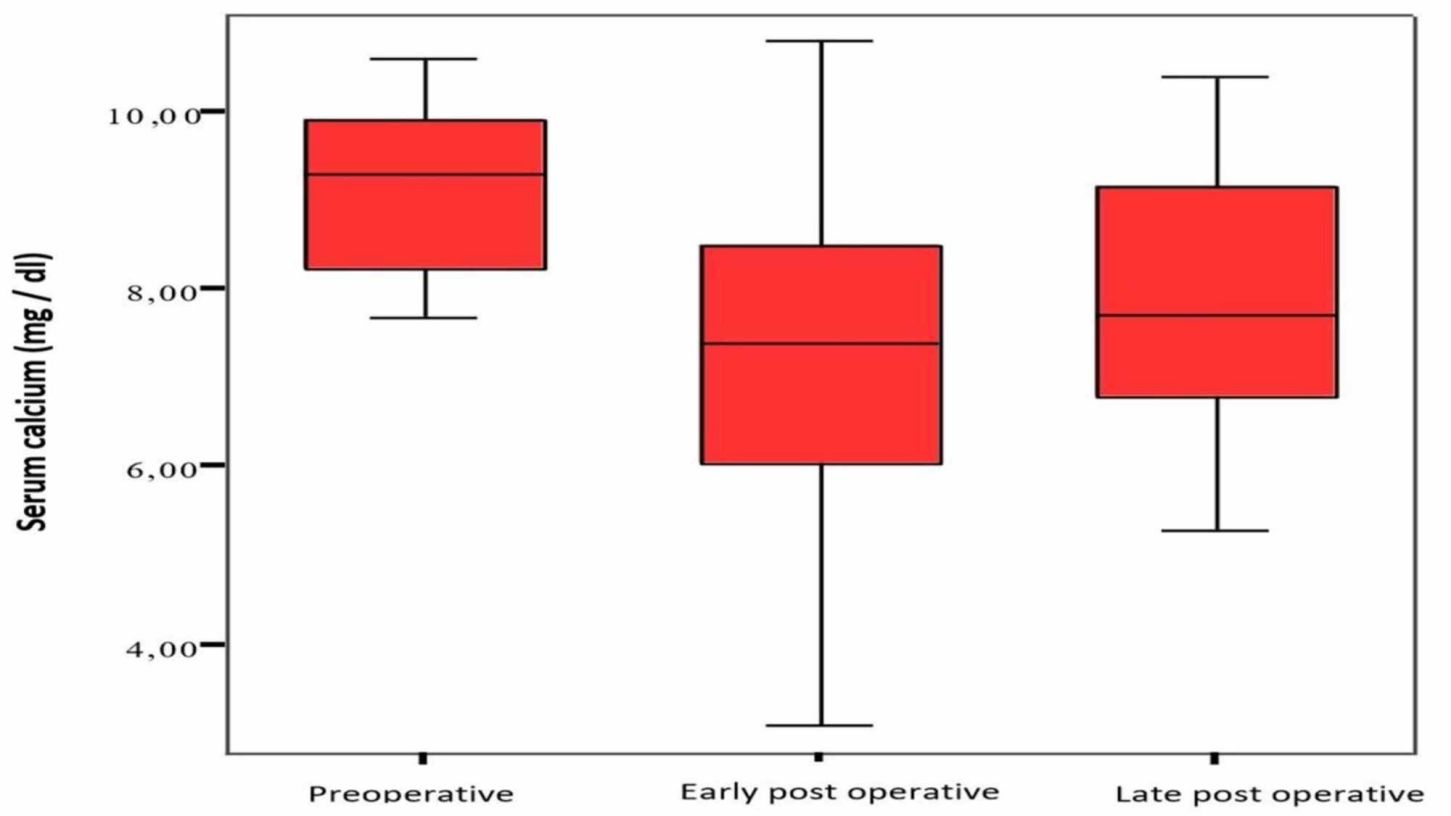
Serum calcium levels Comparison of serum calcium values in the preoperative, early, and late postoperative periods (normal range: 8.40-10.20 mg/dl)

The laboratory values are as follows: serum calcium values: 9.0±1.4 mg/dl in the mean preoperative period, 7.2±1.9 mg/dl in the early postoperative period, and 7.9±1.6 mg/dl in the postoperative period; serum phosphorus values: 7.3±2.2 mg/dl in the mean preoperative period, 6.0±2.1 mg/dl in the early postoperative period, and 5.9±2.3 mg/dl in the postoperative period; and serum PTH values: 2145±910 pg/ml (734-4540 pg/ml) in the mean preoperative period, 243±287 pg/ml (6.5-910 pg/ml) in the early postoperative period, and 470±696 pg/ml (14-2330 pg/ml) in the late postoperative period. When preoperative, early, and late postoperative values were compared, a decrease in serum PTH level and calcium level was found, which was statistically significant (p <0.05). Figure 1 shows the preoperative, early, and late (1 week - 6 months) postoperative period comparisons of serum calcium values.

**Figure 2:**
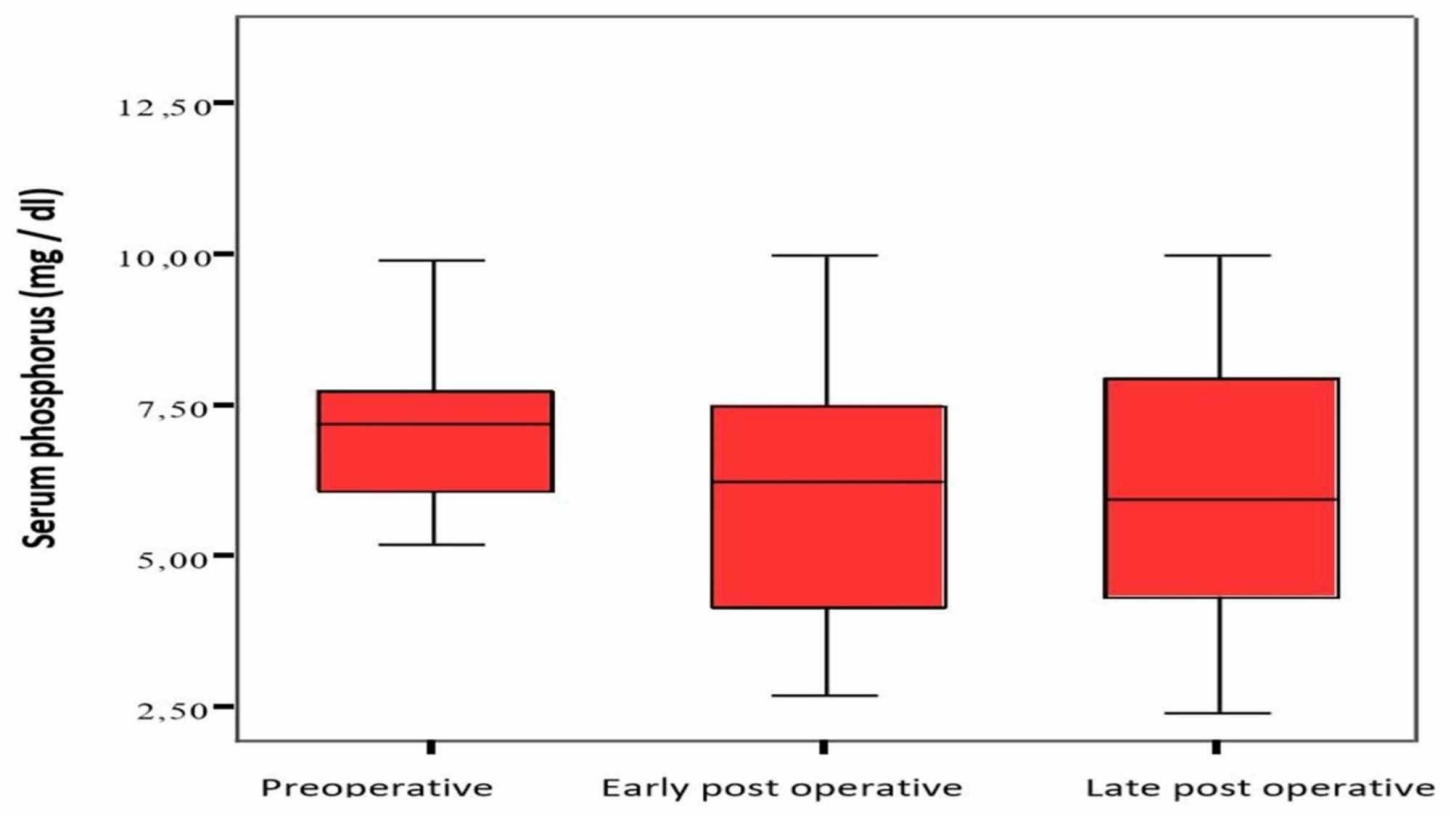
Serum phosphorus level Comparison of serum phosphorus values in the preoperative, early, and late postoperative periods (normal range: 2.70-4.50 mg/dl)

Figure 2 shows the preoperative, early, and late postoperative comparisons of serum phosphorus values.

**Figure 3:**
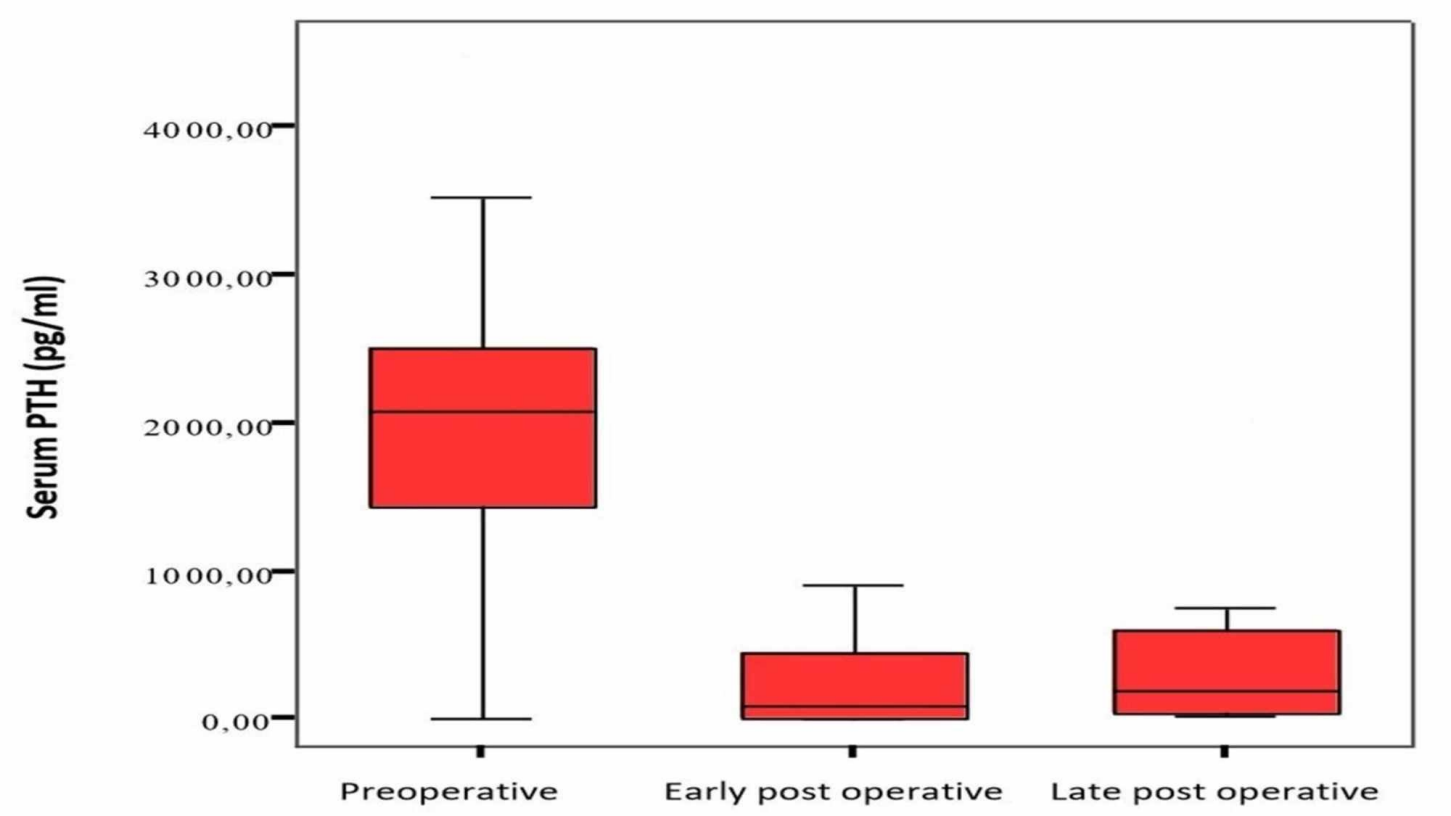
Serum PTH levels Comparison of serum PTH values in the preoperative, early, and late postoperative period (normal range: 10-70 pg/ml)

Figure 3 shows preoperative, early, and late postoperative comparisons of serum PTH values.

Histopathological assay

Histopathological examination revealed parathyroid hyperplasia in all patients.

## Discussion

The most effective and safe treatment method for patients with secondary hyperparathyroidism is total parathyroidectomy and parathyroid autotransplantation [14-15]. The first successful parathyroidectomy was performed in 1928 by Isaac Y. Olch for bilateral parathyroid adenoma in America and later in 1932 by Cope and Churchill for bilateral neck exploration [5]. In times when the frozen procedure is limited, there is no preoperative evaluation and there are no intraoperative assistive techniques, the surgeon's experience is undoubtedly the only determinant for the success of the surgery.

The most commonly used radiological method for imaging parathyroid pathologies is the US. The accuracy rate of localizing adenomas is as wide as 34%-92% because it is dependent on the person performing the US procedure [3,16]. Normal parathyroid glands may not be displayed with the US. It is also difficult to visualize ectopic parathyroid pathologies [5,17]. CT and MRI are not routinely used in preoperative investigations. CT was not performed in any of our cases. Neck MRI was performed one of our cases because of the US and scintigraphy of the neck did not clearly distinguish between parathyroid pathology. However, no additional information was obtained from the US and scintigraphy. Therefore, in the diagnosis of these patients, it is not necessary to use these tests in routine applications because of the high radiation load on CT and the need for anesthesia, especially in younger ages, and does not provide any additional information in addition to other tests.

In parathyroid adenomas, the pathological tissue is removed. In parathyroid hyperplasia, three glands and half of the fourth gland are removed or four glands are removed and parathyroid autotransplantation is performed. When secondary surgery is required due to recurrence, it is more difficult to find the remaining parathyroid gland due to adhesions due to the first surgery. The parathyroid gland is preferred primarily for autotransplantation with the sternocleidomastoid muscle, forearm muscles, or subcutaneous injection. Bilateral neck exploration was performed in all of our cases. In seven patients, three parathyroid glands were removed and no autotransplantation was performed. Zaraca et al. reported that 19 patients with secondary hyperparathyroidism underwent total parathyroidectomy and autotransplantation, the symptoms improved, and recurrence rates were low [18].

Surgical vital dyes have been introduced for the localization of the parathyroid glands a long time ago. Among these, the most well-known are Toluidine-Blue-o and methylene blue. However, Toluidine-Blue-o can lead to serious toxic effects, and that’s why it cannot enter routine use [19]. Although there are successful publications about methylene blue in the localization of parathyroid pathology in the literature, it has not been used routinely yet [20-21].

The most widely used method for parathyroid scintigraphy is Tc-99m-MIBI dual-phase parathyroid scintigraphy. Total uptake depends on mitochondria activity, gland size, and blood flow in the hyperplasic parathyroid glands or adenomas [4]. Single-photon emission computerized tomography (SPECT) improves the separation of focal MIBI retention in parathyroid tissue and thyroid nodules. SPECT is more frequently used in the imaging of ectopic parathyroid glands in the mediastinum region and in recurrent hyperparathyroidism cases [22-25]. Recently, 2-18F-fluoro-2-deoxy-D-glucose (FDG) and positron emission tomography (PET) imaging have been found to be successful in parathyroid pathologies especially in the nuclear oncology field [26].

All of our cases were evaluated with a parathyroid US and parathyroid scintigraphy in the preoperative period. The rate of detection of parathyroid pathologies in both studies was 75% and was consistent with this literature. When the US and scintigraphy were evaluated, parathyroid pathology was detected in 14 of 16 patients (87.5%).

In a study performed by Fuster et al., US sensitivity was reported as 55%, specificity was 67%, scintigraphy sensitivity was 72%, and specificity was 95% [27]. In another study by Ishibashi et al., the sensitivity of the US was reported to be 30% [28]. Kawata et al. found that the correct diagnosis rate was 63.6% in the use of the US to determine the size of the parathyroid glands and preoperative surgery in patients with secondary hyperparathyroidism [29]. In our study and literature, it was observed that the correct diagnosis rates with US were quite wide.

Ubhi et al. have first presented gamma probe guidance for parathyroidectomy in the literature [9]. Tc-99m-MIBI is the most commonly used radiopharmaceutical in the detection of parathyroid glands intraoperatively. Three hours before the operation, a high dose of 15 mCi or immediately before the surgery, a low dose (1 mCi) Tc-99m-MIBI injection is performed. When ex-vivo counting of the excised lesion and ground activity counts are equalized, it is assumed that the lesion is removed correctly [30]. We used a gamma probe in 11 of our patients during surgery. While four (72.7%) of these patients had four parathyroid glands, three (27.3%) had three glands. Out of the 16 patients, four (56.2%) had four parathyroid glands and seven patients had three glands. Of the seven patients in whom all parathyroid glands were not found, three of them used gamma probes while in four of them, gamma probes were not used. As a result of the statistical analysis of all patients, the use of gamma probes was not found to be statistically significant in all parathyroid glands (p>0.05). However, seven parathyroid glands were found in all (100%) of seven patients using a gamma probe by a single pediatric surgeon and a single nuclear medicine specialist. The organs adjacent to the parathyroid gland, especially the heart, which holds the radioactive material to a large extent, and the neck and the parathyroid glands are very close, therefore, there are errors in the counts and the surgeon may be directed to different regions. Therefore, we believe that experience in using gamma probes during surgery is important.

After a successful parathyroidectomy, serum calcium levels may decrease due to decreased PTH levels. While the PTH levels of eight of our cases decreased to normal limits, six of them were below 500 pg/ml and two were over 500 pg/ml.

When the preoperative and postoperative calcium and PTH levels of the patients were compared; the decrease in PTH level and the decrease in calcium level were statistically significant (p <0.05). When the treatment methods are examined, it is seen that the best results are obtained by total parathyroidectomy and parathyroid autotransplantation.

Children and adolescents cannot be considered young adults; they are more affected by hyperparathyroidism, they develop growth disorders, and, after surgery, they develop more hypocalcemia than adults. Therefore, children and adolescents should be followed up and treated in pediatric surgery centers with sufficient experience and equipment.

## Conclusions

The clinical and biochemical findings of children with hyperparathyroidism secondary to end-stage renal failure may be treated safely and effectively with total parathyroidectomy and parathyroid autotransplantation. Gamma probe detection during parathyroidectomy will facilitate unnecessary and excessive dissection as well as surgery in atypical parathyroid glands. For this reason, we recommend performing radioactive marking and gamma probe detection during parathyroidectomy.
